# Conserved Metanephric Kidney Development and Genome Methylation in Red-Eared Slider Turtle (*Trachemys scripta elegans*)

**DOI:** 10.3390/jdb14020016

**Published:** 2026-04-07

**Authors:** Bing Jia, Mohamed Milad, Hannah C. Boehler, Adam Guerra, Joshua Mowry, Jessica Hiley, James Kasen Lisonbee, Michael Hafen, Troy Camarata

**Affiliations:** 1 NYIT College of Osteopathic Medicine, Arkansas State University, Jonesboro, AR 72467, USA; bingjia@creighton.edu (B.J.);; 2 Department of Math and Statistics, Arkansas State University, Jonesboro, AR 72401, USA; mmilad@astate.edu; 3 College of Osteopathic Medicine, Baptist University, Memphis, TN 38104, USA; adam.guerra@baptistu.edu (A.G.); joshua.mowry@baptistu.edu (J.M.); michael.hafen@baptistu.edu (M.H.); 4 Department of Biological Sciences, Arkansas State University, Jonesboro, AR 72401, USA; hiley.jessica26@gmail.com

**Keywords:** turtle, methylation, kidney, Six2, nephrogenesis

## Abstract

Mammals and reptiles possess a metanephric kidney as the terminal renal organ for homeostasis of solutes and waste products. The development of the metanephric kidney has primarily been studied in mammalian model systems. Little is known about the conservation of metanephric kidney formation in non-mammalian species such as reptiles. Uniquely, reptiles maintain kidney progenitor cell populations throughout life and continually develop new nephrons, the functional unit of the kidney. The red-eared slider turtle, *Trachemys scripta elegans*, was utilized to investigate the conservation of reptilian metanephric kidney development. The nephron progenitor cell (NPC) marker, Six2, was detected in whole-mount turtle kidneys in a similar pattern to mammals. However, there were differences in progenitor cell niche morphology where turtle NPC populations formed distinct elongated rows instead of the rosette-like morphology found in the mouse. The pattern of NPC populations in the embryonic turtle kidney was maintained in the adult turtle. Whole-genome bisulfite sequencing was performed on cortical tissue containing the NPC populations from adult turtle kidneys and compared to those of adult mice. Significant conservation of gene methylation was detected in adult cortical tissue between the two species, although unique signatures were detected in turtle samples related to DNA repair and β-catenin signaling. This suggests a high level of conservation of metanephric kidney development at the genetic level.

## 1. Introduction

The metanephric kidney is the definitive organ found in adult mammal, avian, and reptile species. During embryogenesis, the metanephric kidney develops from reciprocal interactions between epithelium of the ureteric bud (UB) and mesenchymal progenitor cells termed metanephric mesenchyme (MM; [1]). Signaling from the MM induces the UB to branch, ultimately creating a collecting duct system, while the UB branch tips induce a subpopulation of MM cells to differentiate into nephron epithelium [1,2]. In mammals, the process of kidney development and nephron endowment ceases prior to or just after birth as a result of nephron progenitor cell depletion [2,3,4]. Therefore, mammals are born with a finite number of nephrons, and any increase in total functional capacity of the kidney occurs primarily through cell hypertrophy [5].

In contrast to mammals, many species of reptiles appear to possess continual nephron endowment of the metanephric kidney. An increasing number of glomeruli were detected in post-hatch kidneys of the green iguana (*Iguana iguana*) and Yarrow’s spiny lizard (*Sceloporus jarrovii*) as body size increased [6,7]. In chicken (*Gallus gallus*), glomerular numbers increased up to 12 weeks post-hatch, indirectly suggesting continual nephrogenesis [8]. A more extensive survey of reptiles found examples of continual nephrogenesis in adult animals in most orders of reptiles [9]. Histological examination revealed the presence of progenitor cell-like mesenchyme adjacent to UB-like epithelium and newly formed nephrons. Furthermore, the embryonic nephron progenitor cell marker, Six2, was detected in both juvenile and adult American alligator (*Alligator mississippiensis*) kidneys, identifying the adult progenitor cell pool [9,10]. How reptiles maintain adult kidney progenitor cells, while mammals do not, remains an open question.

DNA methylation regulates gene expression by altering transcription factor binding or recruiting chromatin-modifying proteins [11]. In mice, differential methylation was detected between the kidneys of P0 and adult animals [12]. There was an increase in methylation of P0 kidneys corresponding to the suppression of cell differentiation. Proper DNA methylation is also required for kidney development, as DNA methyltransferase 1 (*Dnmt1*) knock-out mice display significant congenital renal defects, including loss of progenitor cells and premature differentiation, leading to reduced nephron number [12,13]. Therefore, it appears that DNA methylation is important for kidney progenitor cell maintenance, and genomic methylation patterns are dynamic as tissue develops and matures. However, the role of DNA methylation in reptiles, specifically in nephron progenitor cell maintenance, remains to be fully investigated.

Our previous work revealed that the red-eared slider turtle (*Trachemys scripta elegans*), referred to as the turtle, had a high frequency of nephrogenic zones in adult kidneys compared to other reptile species [9]. Therefore, we chose this species as a comparative model system to investigate kidney progenitor cell ontogeny. We used Six2 to detect nephron progenitor cell populations (NPC) in turtle embryonic kidneys and compared the pattern to the localization of adult progenitor cell niches. With the role genome methylation plays in kidney development and maturation, we decided to compare genome-wide methylation patterns between the turtle and the mouse. DNA methylation of adult kidney cortical tissue from the mouse and the red-eared slider turtle was compared using whole-genome bisulfite sequencing. Analysis identified conserved methylated functional pathways and genes, along with unique turtle methylated genes.

## 2. Materials and Methods

### 2.1. Animals

This study was carried out in accordance with protocols approved by the NYIT College of Osteopathic Medicine and Arkansas State University Institutional Animal Care and Use Committee (FY22-23-9, approved 23 September 2022). Twelve-week-old CBA/CaJ (Jackson Laboratory, Bar Harbor, ME, USA) male mice were used for tissue isolation and genomic DNA and total RNA extraction. Adult *Trachemys scripta elegans* (red-eared slider) turtles were obtained from Carolina Biological Supply (two females and one male). Previous work did not suggest sex differences in continual nephrogenesis [9]. Turtle curved carapace length varied between 185 and 233 mm, indicating sexual maturity; therefore, animals were considered adults. Turtle embryos were obtained from Tangi Turtle Farm (Ponchatoula, LA, USA) in compliance with the Louisiana Department of Agriculture and Forestry between 7 and 10 days post-lay. Eggs were incubated as described [14] at 29 °C. Morphological criteria were used for embryo staging as the environment plays a significant role in developmental timing [14].

### 2.2. Nucleic Acid Extraction and Amplification

Genomic DNA was extracted using the Monarch genomic DNA extraction kit (New England Biolabs, Ipswich, MA, USA) following the manufacturer’s instructions, including proteinase K and RNase A incubations. For mice, the outer cortical tissue was dissected in cold PBS, followed by genomic DNA extraction. For adult turtle kidneys, surface convolutions or medullary tissue were dissected in cold PBS, followed by the extraction of genomic DNA.

Total RNA was extracted from turtle metanephric kidneys at stage 25 [14] and from adult kidney surface convolutions. For embryonic tissue, three pairs of kidneys from separate embryos were pooled for each RNA extraction. Mesonephric tissue was thoroughly separated from the metanephros. The Monarch total RNA miniprep kit was utilized following the manufacturer’s instructions, including on-column DNase I treatment (New England Biolabs, Ipswich, MA, USA). We used 0.5 μg of total RNA (minimum 260/280 ratio of 2.0 and minimum concentration of 150 ng/µL) for reverse transcription, and the resulting cDNA was utilized as template for PCR (95 °C for 30 s, 55 °C for 30 s, 72 °C for 30 s × 32 cycles). The following primers were used for amplification of *T. scripta elegans* gene: *Gapdh*: forward 5′ GGCAAAGTCCAGATTGTAG 3′, reverse 5′ ACAAACATAGGTGCATCAG 3′ [15]; *Six1* (XM_034770394): forward 5′ GGCTTCACGCAGGAGCAAGTGGC 3′, reverse 5′ GGTCTACCAAGCTCGAGGTGAG 3′; *Six2* (XM_034766952): forward 5′ ACTTTTGGATTCACCCAAGACG 3′, reverse 5′ TGGAGTCCTGCAAACTATGATGG 3′; *Cited1* (XM_034782479): forward 5′ CATGAAGGACCGCAAAGCAGTG 3′, reverse 5′ TCAGCGGCTGGAAGGGAAATCT 3′; *Fgf20* (XM_034772484): forward 5′ CTTGGAAGGGTTAGGGCAACAG 3′, reverse 5′ GTAGAGTTCTGGAACTCTTTCAGG 3′. Amplicons were resolved for visualization by 2% low-melt agarose. Agarose resolution of gene amplicons was performed from two biological replicates for embryonic and adult turtle tissue. For semi-quantitative RT-PCR, samples were run on a QuantStudio 3 system (Design and Analysis Software V1.6.1, Thermo Fisher Scientific, Waltham, MA, USA) from two biological replicates, with each biological replicate run in triplicate.

### 2.3. Whole Genome Bisulfite Sequencing

Genomic DNA quality (sample degradation) was assessed using a Qubit Fluorometer (Thermo Fisher, Waltham, MA, USA) and by gel electrophoresis before library construction. DNA samples were submitted to Novogene for paired-end 150-base-pair whole-genome bisulfite sequencing using the Illumina NovaSeq 6000 platform (Illumina CASAVA pipeline, San Diego, CA, USA). Sequence quality and adapter trimming were performed using FastQC and fastp. Sequence reads were then aligned to the mouse (GRCm38) or red-eared slider (CAS_Tse_1.0) genome using Bismark (version 0.24.2). CpG sites were defined as regions in the DNA sequence where a cytosine (C) was followed by a guanine (G) in the 5′ to 3′ direction. Clusters with a high frequency of CpG sites, regions having a length >200 bp, G+C content >50%, and an observed/expected CpG ratio of >0.6, were identified as CpG islands. Differentially methylated cytosines (DMCs) were cytosine bases in the genome that showed a significant difference in methylation levels between test and control samples (adult mouse kidney cortex). Differential DNA methylation was calculated by comparing the proportion of methylated cytosines (Cs) in CpG, CHH, or CHG contexts in a test sample relative to a control using the R methylKit package (release 3.22) [16]. MethylKit performed Fisher’s test on the pooled sample and control groups, and a false discovery rate (FDR)-corrected *p*-value threshold of ≤0.05 was considered significant for identifying differentially methylated cytosines (DMCs). For DMC calculation, the following parameters were implemented:

- win.size: window size of 1000 base pairs;
- step.size: step size of 1000 base pairs;
- cov.bases: minimum coverage of 10 bases.

For complete details on methylKit, please refer to the link: https://bioconductor.org/packages/release/bioc/vignettes/methylKit/inst/doc/methylKit.html (last accessed on 4 January 2023). The following formulas were utilized for bioinformatics: G+C content = [(number of Cs) + (number of Gs)]/length of the window, Observed/Expected ratio = number of CpGs/[(number of Cs * number of Gs)/length of the sequence]. Sequencing data from turtle or mouse were pooled to create lists of methylated genome sites.

### 2.4. Whole-Mount Immunohistochemistry

Embryonic kidneys were dissected at selected stages and fixed in 4% formaldehyde made from paraformaldehyde. Fixed tissue was washed in PBS at room temperature, then dehydrated in a methanol/PBS series followed by storage at −20 °C. For staining, tissue was rehydrated at room temperature in a PBS plus 0.01% tween (PBST) series (75%, 50%, 25%, 0% methanol, 5 min each). Older kidney tissue (stage 23 and older) contained pigmentation and was bleached using 6% H_2_O_2_ in PBST for 1 h at room temperature. Tissue was treated with proteinase-K (10 µg/µL) followed by washes in PBST. Primary antibody was diluted in PBST, containing 1% BSA and 0.5% normal goat serum, and incubated with embryonic kidneys at 4 °C overnight. Primary antibody solution was washed thoroughly with three 1 h washes with PBST while rocking. Secondary antibody was diluted in PBST containing 1% BSA and incubated with tissue overnight at 4 °C, followed by three 1 h washes with PBST while rocking. DAB staining solution was prepared according to the manufacturer’s instructions (BioLegend, San Diego, CA, USA) and incubated with the tissue for 30 min. Dilute H_2_O_2_ was added to enhance chromogen staining. Staining reactions were stopped by washing in PBST with 0.04% sodium azide. Primary antibodies, anti-Six2 (Proteintech, 11561-1-AP, Rosemont, IL, USA) and anti-pan-cytokeratin (BioLegend, clone C-11), were used at a dilution of 1:100. Secondary HRP-conjugated antibodies (BioLegend, anti-rabbit Poly4064; anti-mouse Poly 4053) were used at a 1:250 dilution. A minimum of three kidneys from separate embryos were subjected to immunohistochemistry for each stage and antibody.

### 2.5. Whole Kidney Imaging

Intact renal organs were dissected from embryos in cold PBS with the mesonephros and metanephros attached. Tissue was imaged using an Olympus CX23 fitted with a TrueChrome 4K Pro camera (Olympus, Center Valley, PA, USA). Craniocaudal and mediolateral measurements were conducted in ImageJ (1.54g; Java 1.8.0_345 [64-bit]) using relative pixel counts. The ratio of axes was determined by dividing the average mesonephric measurement by the average metanephric measurement. Kidney tissue from five different embryos was measured for both the mesonephros and metanephros.

## 3. Results

### 3.1. Localization of Adult Kidney Progenitor Cell Populations in Turtles Is Established During Embryogenesis

**Figure 1:**
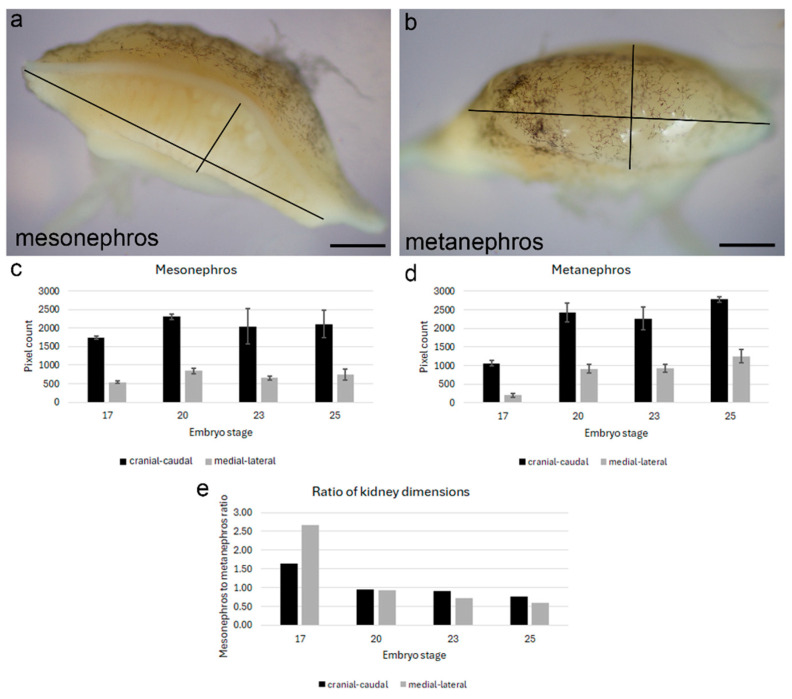
Relationship of renal organ size during embryonic development. (**a**,**b**) Representative craniocaudal (long axis) and mediolateral (short axis) axes at stage 23 of mesonephric (**a**) and metanephric (**b**) kidneys. Scale bars = 50 µm. (**c**,**d**) Average lengths of craniocaudal and mediolateral axes for mesonephric (**c**) and metanephric (**d**) kidneys at different developmental stages. Single kidneys were measured from five independent embryos for each stage. Error bars represent standard deviation. (**e**) Mesonephros to metanephros ratio of craniocaudal and mediolateral axis measurements. Average lengths for the mesonephros were divided by average lengths for the metanephros for each axis.

Our previous work had detected regions of continual nephrogenesis on the periphery of the adult turtle kidney [9]. To better identify these regions and whether they are an extension of embryonically derived nephron progenitor cells, we characterized Six2 protein expression during kidney organogenesis. A developmental series of turtle embryos was collected following previously published guidelines and staging based on morphology [14]. To determine the dynamics of renal tissue growth during development, we measured the craniocaudal axis and the mediolateral axis for both the mesonephros and metanephros (Figure 1a,b). Previous work has shown the turtle kidney to be positioned primarily in the craniocaudal and mediolateral orientation in adults [17]. During the developmental series examined, both kidney types grew in the craniocaudal and mediolateral axes (Figure 1c,d). However, the ratio of axis length shifted from the mesonephros being larger at stage 17 to the metanephros becoming larger by stage 25 (Figure 1e).

**Figure 2:**
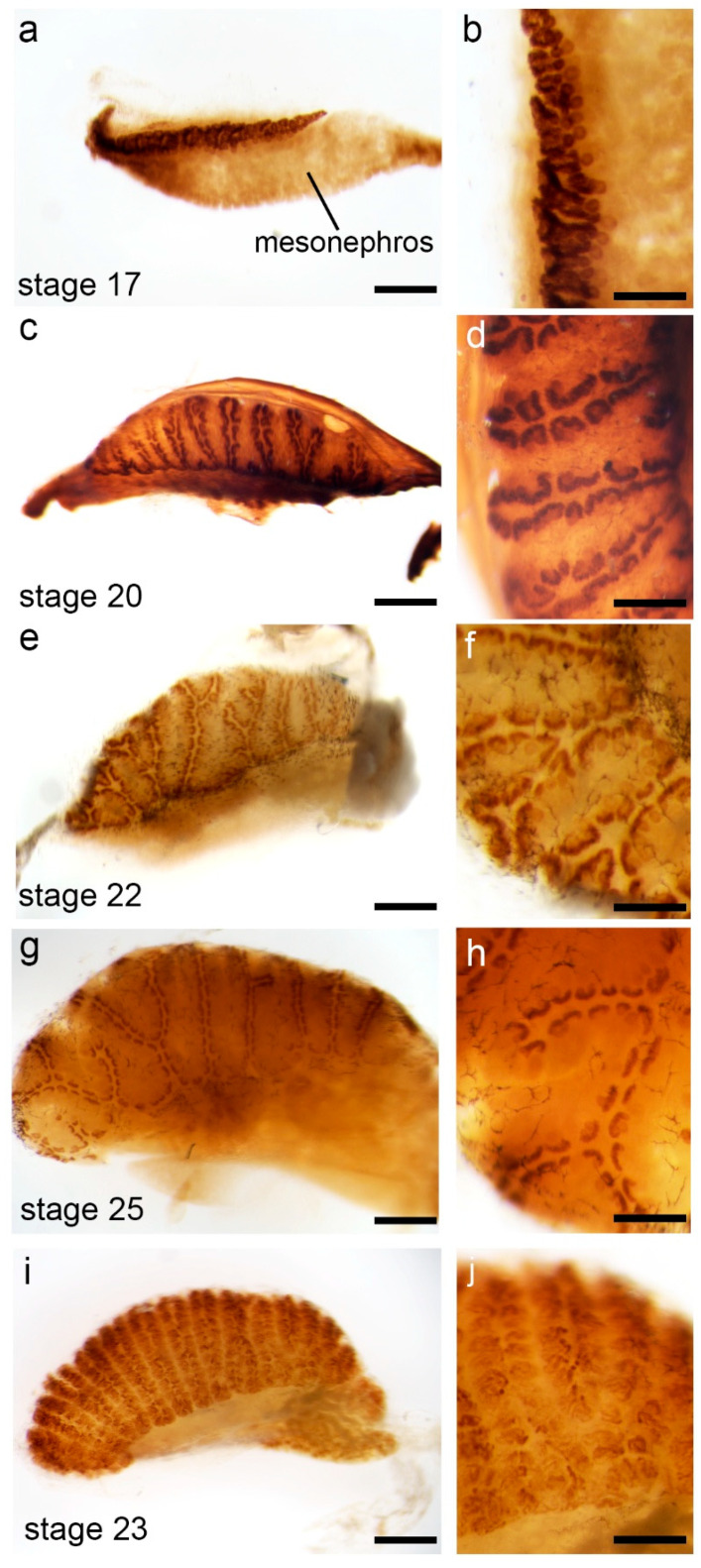
Six2 whole-mount immunohistochemistry. (**a**–**h**) Representative Six2 whole-mount immunohistochemistry of different embryonic stages. (**b**,**d**,**f**,**h**) Zoomed portions of images on the left. (**i**,**j**) Cytokeratin whole-mount immunohistochemistry of stage 23 embryonic kidney. (**j**) Zoomed portion of image in (**i**). Representative images are shown. A minimum of 3 kidneys from separate embryos were subjected to immunohistochemistry for each stage and antibody. Scale bars in (**a**,**c**,**e**,**g**,**i**) = 50 µm; scale bars in (**b**,**d**,**f**,**h**,**j**) = 25 µm.

Next, kidney tissue was subjected to whole-mount antibody staining for Six2 (Figure 2). Six2-expressing NPCs in the metanephric kidney were detected in all stages examined. No expression of Six2 was detected in the mesonephros, suggesting no active nephrogenesis in this tissue (Figure 2). As the metanephros grew, Six2-expressing cell populations became more defined as half-circles arranged in distinct rows along the surface of the developing organ. The tissue between the Six2-expressing progenitor cells appeared to contain differentiated epithelium based upon staining with a pan-cytokeratin antibody (Figure 2i,j). The expression of Six2 in nephron progenitor cells of the developing turtle kidney was similar to what has been reported in the mouse, suggesting evolutionary conservation [10,18]. However, the overall morphology of Six2-expressing progenitor cell populations was unique in the developing turtle kidney. Condensed groups of progenitor cells in the turtle were spread out along the surface of the developing kidney and aligned in rows. This is in contrast to the highly compact, rosette-like organization of nephron progenitor cells on the surface of the embryonic mouse kidney [2].

**Figure 3:**
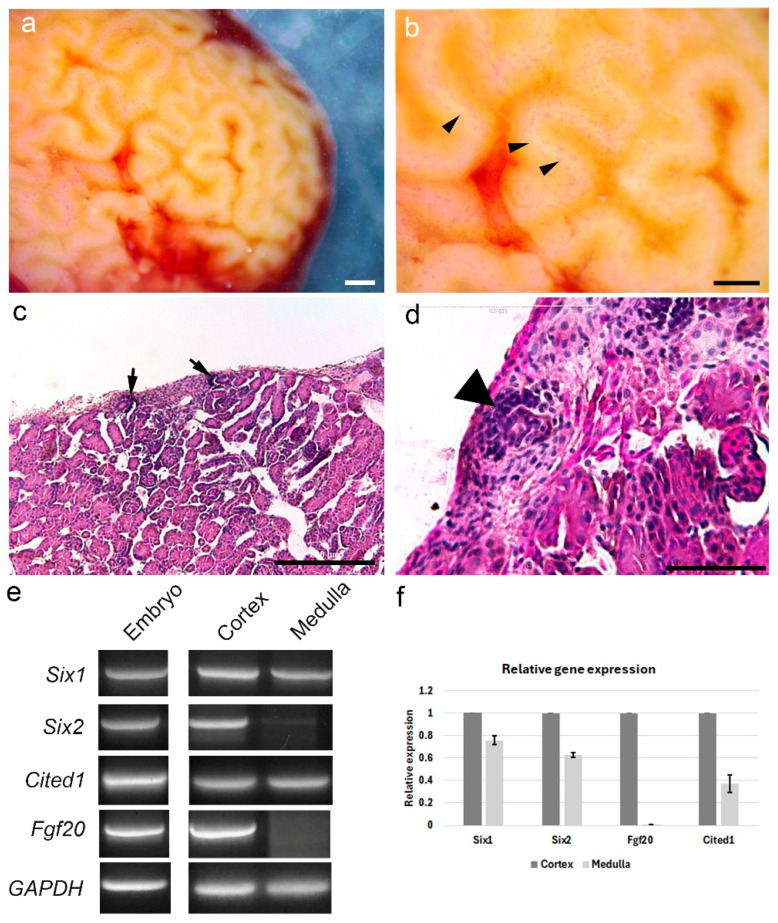
Zones of continual nephrogenesis on the surface of the adult turtle kidney. (**a**) Gross image of an adult turtle kidney showing surface convolutions. Scale bar = 1 mm. (**b**) Zoomed portion of surface convolutions showing parallel lines of renal corpuscles (arrowheads) where nephron progenitor cells are located. Scale bar = 1 mm. (**c**) Section histology of adult turtle kidney showing zones of continual nephrogenesis from the parallel lines on the surface of the kidney (arrows). Scale bar = 200 µm. (**d**) Higher magnification showing a zone of nephrogenesis consisting of condensed mesenchyme adjacent to a ureteric bud-like epithelial tubule (arrowhead). Scale bar = 50 µm. (**e**) Expression analysis of nephron progenitor genes comparing embryonic kidney to expression in the adult kidney cortex or medulla. (**f**) Relative gene expression using semi-quantitative PCR for genes shown in (**e**). Gene expression in the medulla is shown relative to the cortex. Note: the original scale bar in image (**d**) appears at the top of the image.

We wished to determine if we could use the embryonic kidney progenitor cell population morphology as a template to identify regions of nephrogenesis on the surface of the adult turtle kidney. The surface of the adult turtle kidney was found to be highly convoluted as previously described (Figure 3a,b; [17]). Along the convolutions, parallel rows of red spots could be detected, most likely indicating capillary vessels within renal corpuscles. We suspected this was the region where adult kidney progenitor cells may reside based upon our previous work with continual nephrogenesis in reptiles [9]. For example, in the American alligator (*Alligator mississippiensis*), regions of adult continual nephrogenesis could be detected along surface convolutions. To further assess if a similar morphology was found in the turtle, we sectioned and stained kidney tissue to detect regions of continual nephrogenesis (Figure 3c,d). From stained sections, we could observe parallel regions of continual nephrogenesis that fit the morphology detected on the kidney surface. Higher magnification showed the dense, dark staining nephron progenitor cells adjacent to an epithelial tubule, similar to what has previously been observed in reptiles (Figure 3d; [9]).

To further determine if the surface convolutions of the adult turtle kidney contained regions of continual nephrogenesis, we dissected the surface convolutions for total RNA extraction. cDNA was then created for the purpose of detecting kidney developmental gene expression (Figure 3e,f; see Section 2. Materials and Methods). For comparison, cDNA was reverse-transcribed using RNA extracted from adult turtle kidney medulla, deeper from the surface convolutions, and from the developing metanephric kidney. The genes tested could all be detected in the embryonic metanephric kidney from the turtle, as expected (Figure 3e). However, unique expression patterns were found when comparing the surface convoluted regions containing the regions of continual nephrogenesis and deeper medullary cells. While all genes tested were detected in cortical tissue of the surface convolutions, similar to the embryonic kidney, *Six2* and *Fgf20* were reduced or not detected from deeper medullary cells as detected by gel electrophoresis (Figure 3e). Relative gene expression using semi-quantitative RT-PCR (qPCR) further supported differences between the cortex and medulla (Figure 3f). All of the genes tested showed reduced levels of expression in the medulla as compared to the cortex of the adult turtle kidney. The largest difference in expression was detected for *Fgf20*, which showed little to no expression through qPCR in the medulla (Figure 3f). In total, these data suggest that the locations of adult continual nephrogenesis are established during embryonic development and are most likely a continuation of the embryonic program.

### 3.2. Whole Genome Bisulfite Sequencing Identifies Common Pathways Between Mouse and Turtle

**Figure 4:**
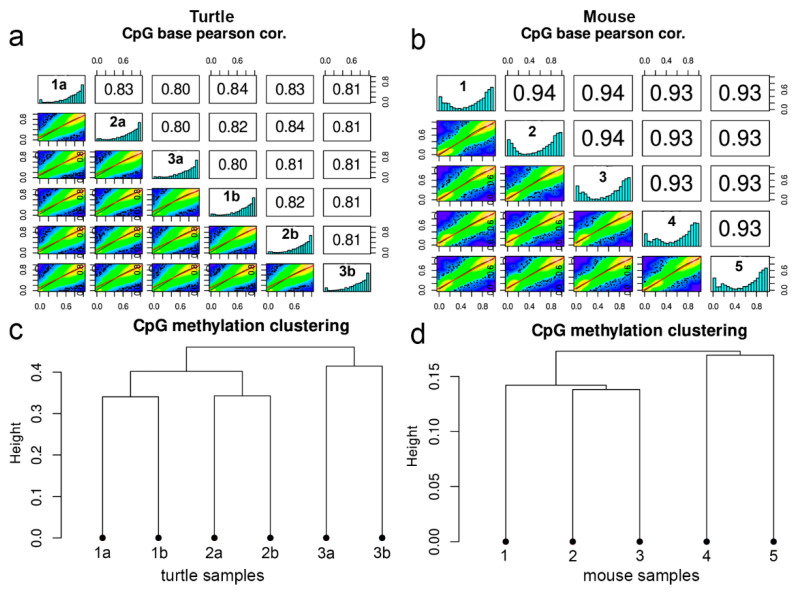
Whole-genome bisulfite sequencing sample comparison. (**a**,**b**) Pearson’s correlation analysis of individual whole-genome bisulfite sequencing (WGBS) samples from adult turtle kidney tissue (**a**) and adult mouse kidney tissue (**b**). For adult turtle kidney tissue, two independent samples were collected from each animal (i.e., animal 1a, animal 1b). (**c**,**d**) Methylated sample clustering between turtle (**c**) and mouse (**d**). For turtles, dissection replicates from the same animal clustered together.

We next decided to determine if there were epigenetic differences between the adult turtle and mouse kidney. Utilizing the ability to detect and isolate regions of continual nephrogenesis from the surface convolutions of the adult turtle kidney, we extracted genomic DNA for the purpose of whole-genome bisulfite sequencing (WGBS). Genomic DNA was extracted from three different adult turtles with two independent dissections from each animal (thus three biological replicates with two technical replicates per animal) to help account for differences in tissue dissection. For comparison, genomic DNA was extracted from the kidney cortical tissue of five different adult mice, which was then subjected to WGBS. The tissue organization of the kidney cortex is similar between mouse and turtle and includes renal corpuscles as well as straight and convoluted tubules [9]. Quality control statistics for both turtle and mouse WGBS are presented in Supplemental Data. Pearson’s correlation coefficients were higher in mouse than in turtle samples, yet turtle samples still showed strong correlations (Figure 4a,b). For the turtle, sample methylation profiles clustered with each dissection replicate from an individual animal (Figure 4c). This suggested that differences in surface convolution dissection did not appreciably contribute to differences in methylation profiles. The mouse samples showed close methylation clustering (Figure 4d).

**Figure 5:**
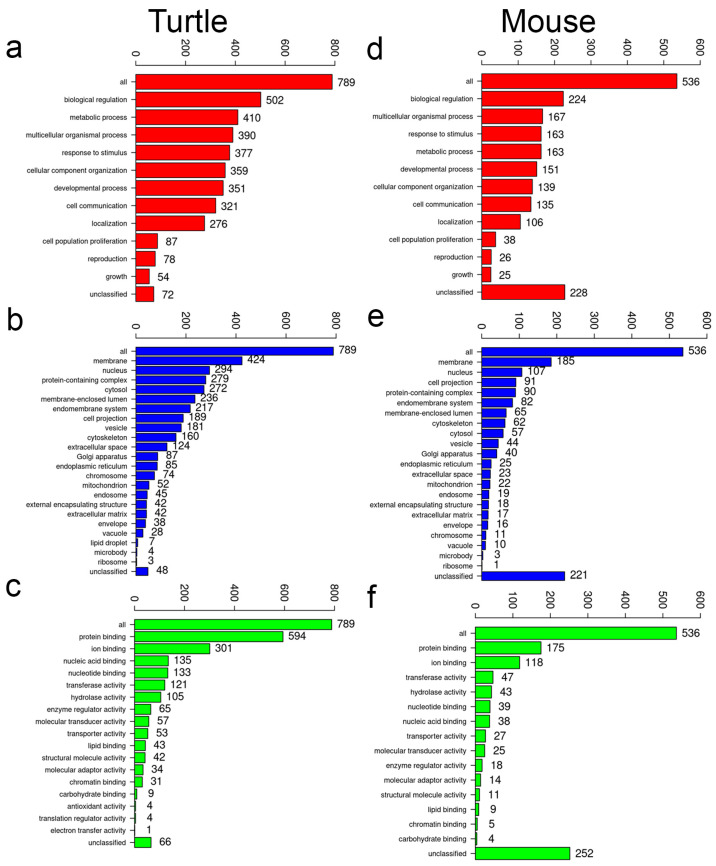
Gene ontology analysis of intragenic methylated gene sets of turtle and mouse genomes. (**a**–**c**) Turtle intragenic methylation GO subset analysis of biological process, cellular component, and molecular function, respectively. (**d**–**f**) Mouse intragenic methylation GO subset analysis of biological process, cellular component, and molecular function, respectively.

Identified methylation sites were classified as upstream, within (intragenic), intergenic, or downstream of a gene. Methylation site comparisons showed a larger number of sites in all classifications from the turtle genome compared to the mouse (Table 1). However, the number of methylation sites that corresponded to unique genes was more variable. Unique genes identified in the methylation analysis for mouse and turtle are found in the Supplemental Data. Upstream, intragenic, and downstream gene sets were subjected to gene ontogeny (GO) subset analysis for biologic, cellular, and molecular function (Figure 5; Supplemental Figures S1 and S2) [19,20]. Similar GO terms were identified in all three classifications of methylation sites in turtle and mouse gene sets. To gain further insights, upstream, intragenic, and downstream gene sets were analyzed for GO gene over-representation analysis [19,20]. The upstream and downstream gene sets did not identify any over-represented pathways that had a false discovery rate (FDR) ≤0.05, the cutoff for significance. A lone exception was the uronic acid metabolism process GO term in the upstream mouse gene dataset. The intragenic classification of methylated genes was much larger for both turtle and mouse compared to the other methylation classifications (Table 1). Over-representation analysis of this gene set found similar processes and functions such as synapse structure and function, cell projection, cell adhesion, and neuron differentiation (Supplemental Figure S3; Table 2). However, the turtle samples did reveal unique terms not detected in the mouse related to small GTPase signaling, DNA repair, and β-catenin signaling (Supplemental Figure S3; Table 2).

**Figure 6:**
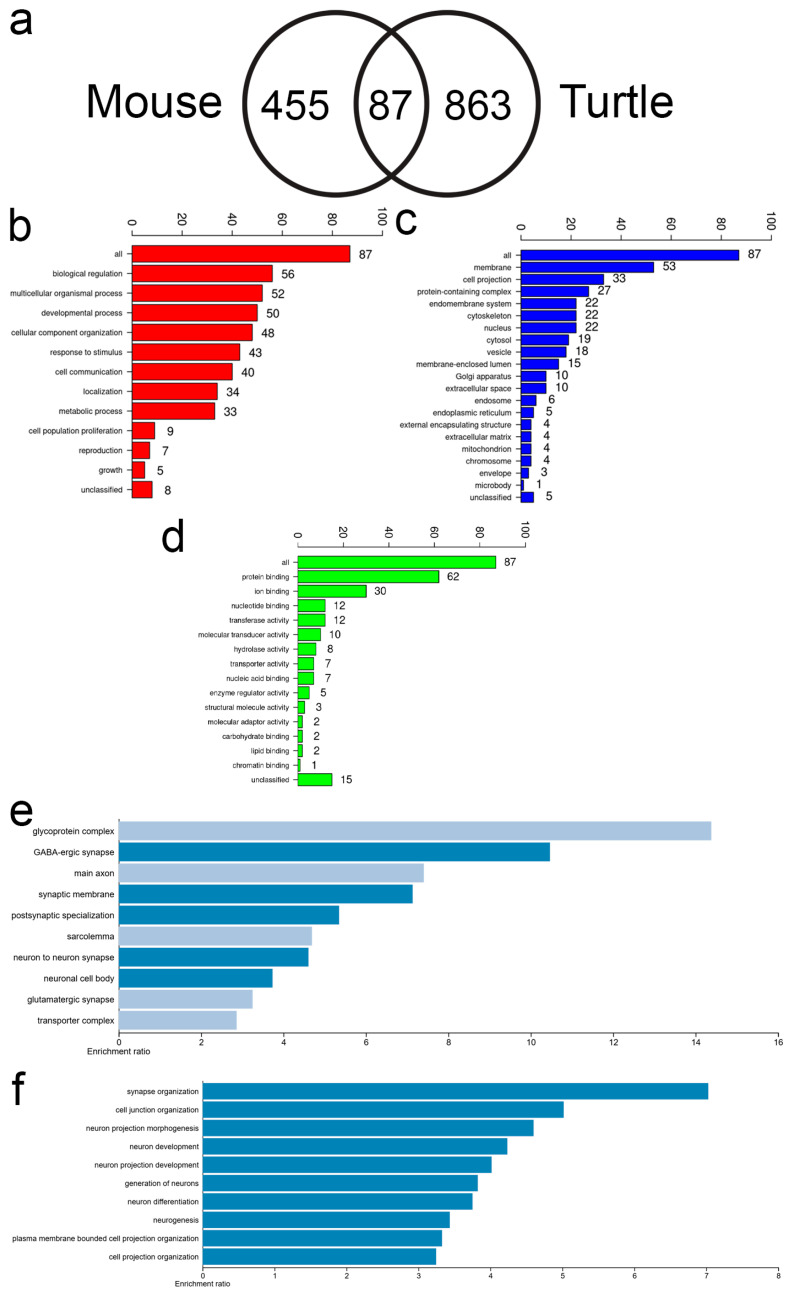
Gene ontology and over-representation analysis of common methylated genes. (**a**) Venn diagram of intragenic methylated genes from turtle and mouse. A total of 87 methylated genes were shared between adult turtle and mouse gene methylation. (**b**–**d**) GO subset analysis of biological process, cellular component, and molecular function, respectively. (**e**,**f**) Over-representation analysis of common genes for biological process (**e**) and cellular component (**f**). Analysis of molecular function did not yield any features with a false discovery rate (FDR) ≤ 0.5. (**e**,**f**) Dark blue bars represent FDR < 0.5 and light blue bars represent FDR > 0.5.

We next directly compared the intragenic methylated gene sets from each species and found a common subset of 87 genes (Figure 6a; Supplemental Data). GO subset analysis showed a majority of the shared methylated genes to be involved in biological regulation, multicellular organismal process, and developmental processes (Figure 6b–d). Gene over-representation analysis found strong relationships in processes related to neuron development and differentiation, synapse organization, and cell junction organization (Figure 6e,f). There were no common genes identified with upstream or downstream methylation sites between turtle and mouse. The methylation data suggest conservation of intragenic methylation with respect to specific genes or in functional pathways related to neurogenesis. Some unique features were detected in the turtle samples, which may relate to the NPC function, such as β-catenin and cytoskeleton activity.

## 4. Discussion

### 4.1. Embryonic Kidney Development and Unique Morphology of Nephron Progenitor Cell Populations

During development, we observed changes in renal organ size. The mesonephros and metanephros expanded in the craniocaudal axis as well as the mediolateral axis between stages 17 and 20. While the mesonephric kidney did not appreciably grow between stages 20 and 25, the metanephros continued to expand. This was evident by the changes in the ratio of axis length between the mesonephros and metanephros. At stage 17, the mesonephros was 1.7 and 2.6 times longer than the metanephros in the craniocaudal and mediolateral axes, respectively. However, the ratio was reversed by stage 25, where the metanephros was almost twice the length of the mesonephros in the craniocaudal and mediolateral axes. The maintenance of the mesonephros and dynamic changes in metanephric kidney size align with observed kidney organ allometry in lizards [7]. Neither the mesonephric nor the metanephric kidney mass correlates with body size individually. However, together the two renal organs show an expected relationship between renal mass and body mass [7]. The mesonephros remains functional post-hatch in lizards until the metanephros is of sufficient size [7]. It is likely that a similar mechanism occurs in turtles, and the establishment of renal organ size differences occurs during embryonic development.

In mammals, the transcription factor Six2 marks the metanephric kidney NPCs [10]. Six2 expression was found to be conserved in the developing metanephric kidney of the red-eared slider turtle (Figure 1). Pools of Six2^+^ progenitor cells were observed in parallel rows on the periphery of the developing turtle kidney, with the intervening space presumably filled by cytokeratin-positive epithelium. The presence of a Six2^+^ mesenchyme cap adjacent to ureteric bud epithelium is an expected and conserved finding based on studies with human and mouse embryonic kidneys [2,21,22]. However, in mammals, the overall morphology of nephron progenitor cell pools was commonly in a rosette-like pattern [22]. In the turtle, the nephron progenitor cell pools were localized in distinct rows that branched as development proceeded. Comparing the embryonic and adult turtle kidney, it appears that the row-like organization of nephron progenitor cells of the embryonic kidney established the adult convolutions containing zones of continual nephrogenesis. A similar progenitor cell localization was found in the adult kidney of the American alligator, where zones of nephrogenesis aligned with surface convolutions [9]. In the American alligator, the rows of continual nephrogenesis at the apex of each renal lobe established the highly organized microanatomy of the kidney [9,23]. It is unknown if the unique morphology of nephron progenitor cells in the turtle has any functional implications. In mammals, over half of nephrons are formed during the final stages of nephrogenesis when nephron progenitors and ureteric bud epithelium are packed into the rosette-like orientation [2,22]. The mammalian NPCs become depleted as differentiation overcomes self-renewal. In the turtle, the NPCs are maintained. It is possible that the rows of nephron progenitor cells in reptiles allow for slower continual growth, while the compact rosette-like orientation in mammals is needed for the concentrated burst of nephrogenesis. It will be of interest to compare the proliferation dynamics of progenitor cells in the developing and post-hatch turtle in relation to progenitor pool morphology.

### 4.2. Differential Gene Expression Between Cortical and Medullary Regions of the Turtle Kidney

Previous work detected the expression of Six2 in zones of continual nephrogenesis in the American alligator, suggesting that adult progenitor cells may be a continuation of an embryonic program [9]. To gain further insight into the embryonic-like state of nephrogenic zones in the adult turtle, we assessed the expression of *Six1*, *Six2*, *Cited1*, and *Fgf20*, genes known to mark and play a functional role in nephron progenitor cell maintenance [1]. Gene expression was compared between the surface convolutions containing regions of continual nephrogenesis and deeper kidney tissue, which possess more differentiated cells. As expected, all genes were detected in embryonic kidneys from turtles (Figure 2). However, differences in gene expression were detected between cortical and medullary tissue. All four genes examined showed reduced expression in the medulla compared to the cortex, with *Fgf20* appearing to be absent from the medullary tissue. *Six1*, *Six2, Cited1*, and *Fgf20* are important for embryonic nephron progenitor cell maintenance in mammals, and a reduction in expression has led to renal dysplasia [10,24,25,26,27]. These genes have not been detected in mouse or human adult kidney tissue [9,28]. The limited differential gene expression supports the hypothesis that adult reptile continual nephrogenesis is an extension of embryonic nephrogenesis. It may be likely that the four genes examined play similar roles in the adult turtle kidney progenitor cell as they do during embryonic development. The small sample size of adult turtles tested in this study is a limitation, and future studies will target a more thorough examination of gene expression differences between the embryonic and adult kidney from both the turtle and the mouse.

### 4.3. Conservation of Methylated Pathways in the Adult Mouse and Turtle Kidney

Conservation of DNA methylation has been shown amongst a large number of vertebrates and tissues [29,30,31,32]. Between different mammals, conserved gene imprinting, methylation valleys, and functional silencing of retroviral elements have been revealed [31]. Furthermore, similar methylation patterns were found between tissues from rats, mice, and humans [30]. A broader species comparison utilizing mouse and zebrafish detected similar levels of increased methylation of gene regions compared to promoter regions between the two species [29]. A significantly larger study of more than 500 vertebrates found that methylation patterns were more similar between organs of different species than between different organs within species [32].

Specific genome methylation patterns in the developing and adult mouse kidney have only recently been investigated [33]. Comparison of newborn mouse kidneys, which still undergo active nephrogenesis, to adult kidneys showed a hypomethylated state in adult ones [12]. Genomes of adult kidney cells had hypomethylated promoters and enhancers associated with differentiation genes, while developmental genes showed enrichment for hypermethylation. This suggested there were specific methylation dynamics between self-renewing progenitor cells and differentiating cells within the kidney [12]. Further support for the importance of genome methylation in the kidney has come from the investigation of mouse DNA methyltransferases [13]. The methyltransferase, *Dnmt1*, was strongly expressed in nephron progenitor cells and their newly formed derivatives but not in more mature cell types or in adult cells. Furthermore, conditional deletion of *Dnmt1* in kidney progenitor cells resulted in reduced organ size with a significant reduction in progenitor cell self-renewal [13].

In the turtle, dissection of cells from regions of continual nephrogenesis revealed a larger number of methylation sites and unique genes compared to the cortical tissue of the adult mouse (Table 1). The *T. scripta elegans* sequenced genome is 2.1 Gb in size compared to the 2.7 Gb size of the mouse genome, further supporting a higher methylation status in our turtle samples (turtle CAS_Tse_1.0 and mouse GRCm38). The increased methylation in turtle cortical tissue aligns with a more undifferentiated state of cells in the surface convolutions from the adult turtle kidney. Although there were differences in total methylation sites detected between the two species, pathway analysis found common cellular and molecular pathways. Additionally, a conserved set of methylated genes between turtle and mouse was identified. Previous work has found that the global genome methylation pattern of turtles and crocodiles was more similar to mammals than to fish or amphibians [34]. Our data support a hypothesis where the pattern of gene methylation in the adult kidney is evolutionarily conserved and was possibly first established in reptiles. Interestingly, the conserved methylated genes and pathways were identified by methylation sites within gene bodies (intragenic). Differential gene body methylation has been observed in human kidneys in the context of fibrosis [35]. The full function of intragenic methylation is unclear, but it may correlate with gene expression [36].

Gene ontology analysis found conservation of intragenic methylated genes in the turtle and mouse kidney cortex related to neuronal cell function and differentiation (Figure 6 and Figure S3). It is unclear the significance of the neuron-related pathways or the functional role of gene methylation in this context. Inhibition of neuronal pathways has been found to be important for the improvement of kidney organoid formation with reduced non-renal cell contamination [37]. Other conserved methylated gene sets related to cell junctions, protein complexes, and cytoskeleton may represent conserved cellular functions utilized by renal cortical cells (Figure S3). Additional experimentation is needed to identify the role of intergenic methylation on gene expression and cellular behavior.

Over-representation analysis did detect unique signatures in the turtle that may be related to the presence of progenitor cell populations. Gene ontology for DNA repair was identified (Table 2). Genes associated with DNA repair have been found to be expressed at higher levels in embryonic kidney progenitor cells in mice compared to adult tissue. Furthermore, intact DNA repair is needed to maintain the genome of kidney progenitor cells during development [38]. Molecular functions for β-catenin were also identified by over-representation analysis. β-catenin is important for kidney development and involved in moving cells from the progenitor cell niche through differentiation [39,40,41]. The β-catenin pathway directly regulates *Fgf20* expression and has been found to interact with Six2 to promote progenitor cell self-renewal [40,42]. Furthermore, Cited1 has been shown to repress β-catenin signaling to help modulate progenitor cell differentiation [43]. The overall functional relationship between the differential gene expression identified in the turtle kidney cortex and gene methylation status remains to be determined.

## 5. Study Limitations

There are limitations to the current study. First, bulk whole-genome bisulfite sequencing may mask individual cell methylation patterns. As the nephron progenitor cells are likely to be a subset of cells isolated from our dissection approach, it is possible that additional functional methylation profiles were missed. Further studies utilizing single-cell resolution approaches will provide additional and valuable information. Second, the limited number of adult turtle samples prevented robust analysis between male and female adults. Even though our previous work did not suggest differences in continual nephrogenesis and maintenance of NPCs between sexes [9], differences between males and females remain possible. Lastly, the GO terms identified in our analysis are interesting observations that require functional experimentation to validate our findings. Future studies will be focused on using the current data as a foundation for functional studies in cells and tissues.

## 6. Conclusions

Our comparative data are supported by previous studies investigating the evolutionary conservation of genome methylation and point toward a highly conserved set of methylated genes and functional pathways in the adult metanephric kidney. Additionally, unique GO terms were identified in the adult turtle kidney cortex that align with the presence of progenitor cell populations. Future studies will focus on single-cell analysis and gene methylation function to further support the current findings.

## Figures and Tables

**Table 1:** Turtle and mouse kidney methylation summary.

	Number of Methylation Sites	
	Turtle	Mouse
Gene (intragenic)	11,233	3407
Upstream	2696	289
Downstream	1895	560
Intergenic	20,518	4778
	Number of unique genes	
	Turtle	Mouse
Gene (intragenic)	1024	560
Upstream	165	110
Downstream	133	148
	Average percent methylation	
	Turtle	Mouse
CpG	74	73
CHG	2.9	2.8
CHH	3.6	3.3

**Table 2:** Methylated gene ontology over-representation analysis.

	Mouse	Turtle
Biological Process		
	▪ presynapse organization	▪ regulation of postsynaptic membrane neurotransmitter receptor levels
	▪ protein localization to the cell junction	▪ neuron migration
	▪ regulation of postsynaptic membrane neurotransmitter receptor levels	▪ protein-containing complex localization
	▪ postsynapse organization	▪ neuron projection guidance
	▪ establishment or maintenance of cell polarity	▪ synapse organization
	▪ cell–cell adhesion via plasma-membrane adhesion molecules	▪ cell–cell adhesion via plasma-membrane adhesion molecules
	▪ regulation of synapse structure and activity	▪ cell junction assembly
	▪ cell junction assembly	▪ axonogenesis
	▪ dendrite development	▪ regulation of neuron projection development
	▪ regulation of membrane potential	▪ small GTPase-mediated signal transduction
Cellular Component		
	▪ glycoprotein complex	▪ DNA repair complex
	▪ ribbon synapse	▪ GABAergic synapse
	▪ GABAergic synapse	▪ sarcolemma
	▪ synaptic membrane	▪ microtubule-associated complex
	▪ adherens junctions	▪ synaptic membrane
	▪ neuron-to-neuron synapse	▪ adherens junction
	▪ postsynaptic specialization	▪ neuron spine
	▪ actin-based cell projection	▪ postsynaptic specialization
	▪ neuron spine	▪ neuron-to-neuron synapse
	▪ transporter complex	▪ glutamatergic synapse
Molecular Function		
	▪ glutamate receptor activity	▪ dynein light intermediate chain binding
	▪ structural constituent of a synapse	▪ phosphotransferase activity, phosphate group as acceptor
	▪ transferase activity, transferring sulfur-containing groups	▪ nucleobase-containing compound kinase activity
	▪ transporter regulator activity	▪ dynein intermediate chain binding
	▪ cell adhesion molecular binding	▪ beta-catenin binding
		▪ cytoskeleton motor activity
		▪ extracellular matrix structural constituent
		▪ nucleoside-triphosphate regulator activity
		▪ protein serine kinase activity
		▪ actin binding

## Data Availability

Whole-genome bisulfite sequencing data have been deposited in the NCBI’s Gene Expression Omnibus and are accessible through accession number GSE18499. All other data and reagents are available upon request.

## Supplementary Materials

The following supporting information can be downloaded at: https://www.mdpi.com/article/10.3390/jdb14020016/s1. Figure S1: Gene ontology analysis of upstream methylated gene sets of turtle and mouse genomes. (a–c) Turtle upstream methylation GO subset analysis of biological process, cellular component, and molecular function, respectively. (d–f) Mouse upstream methylation GO subset analysis of biological process, cellular component, and molecular function, respectively. Figure S2: Gene ontology analysis of downstream methylated gene sets of turtle and mouse genomes. (a–c) Turtle downstream methylation GO subset analysis of biological process, cellular component, and molecular function, respectively. (d–f) Mouse downstream methylation GO subset analysis of biological process, cellular component, and molecular function, respectively. Figure S3: Over-representation gene ontology analysis of turtle and mouse intragenic methylated genes. (a–c) Mouse intragenic methylated gene over-representation for biological process (a), cellular component (b), and molecular function (c). (d–f) Turtle intragenic methylated gene over-representation for biological process (d), cellular component (e), and molecular function (f). Dark blue bars represent false discovery rate (FDR) ≤ 0.5 and light blue bars represent FDR > 0.5. Supplemental Data: supplemental tables representing sequencing quality control data for turtle and mouse and methylation lists with accession numbers of turtle and mouse genes for intragenic (gene), upstream, downstream, and common methylated gene sets.
