# Supplementary figures and images for: Conserved Metanephric Kidney Development and Genome Methylation in Red-Eared Slider Turtle (Trachemys scripta elegans)
Source: J Dev Biol. 2026 Apr 7;14(2):16. doi: 10.3390/jdb14020016 (PMC13108046; doi:10.3390/jdb14020016)

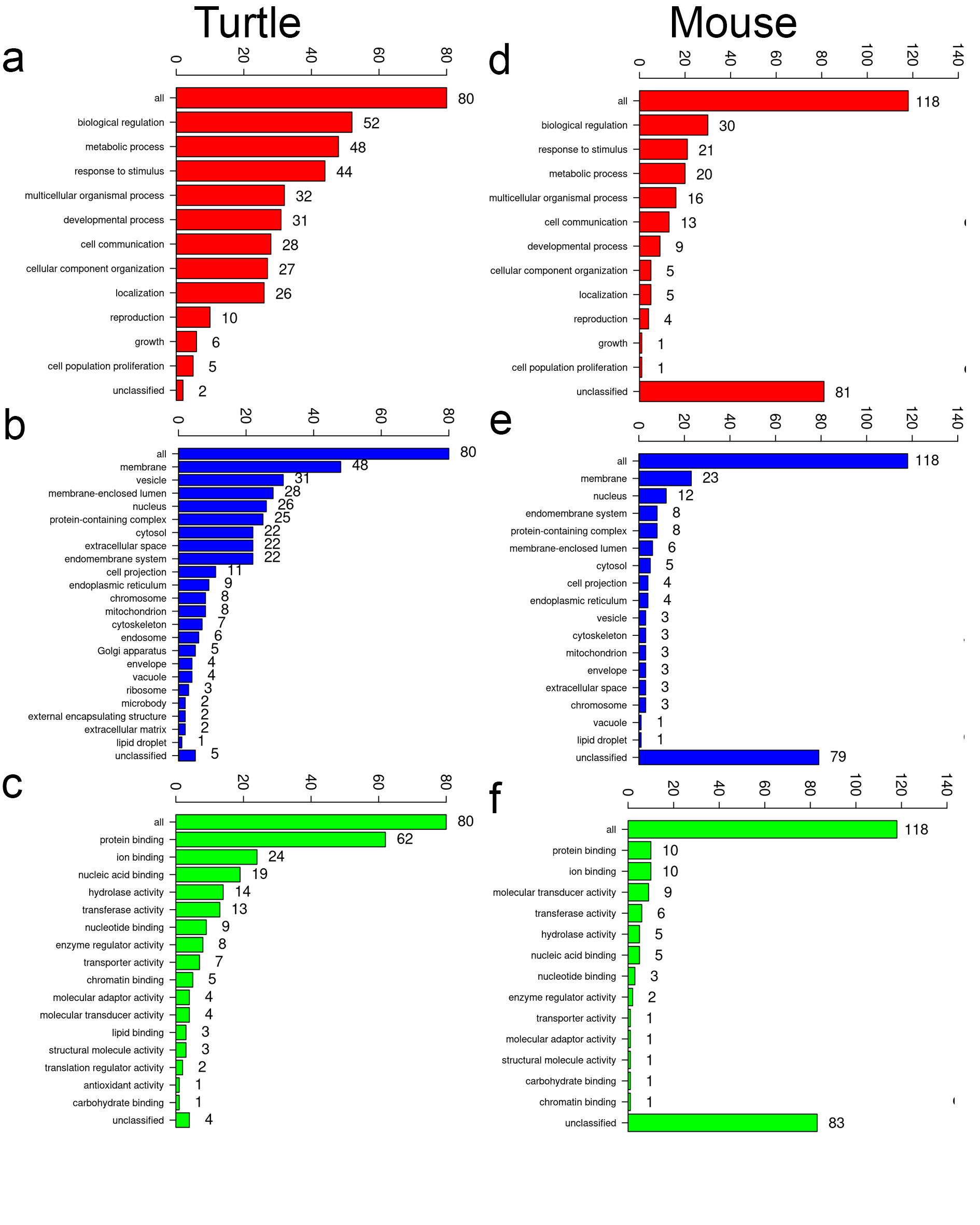

Supplement: Supplementary file 1 [file jdb-14-00016-s001.zip › Figure S1.tif]

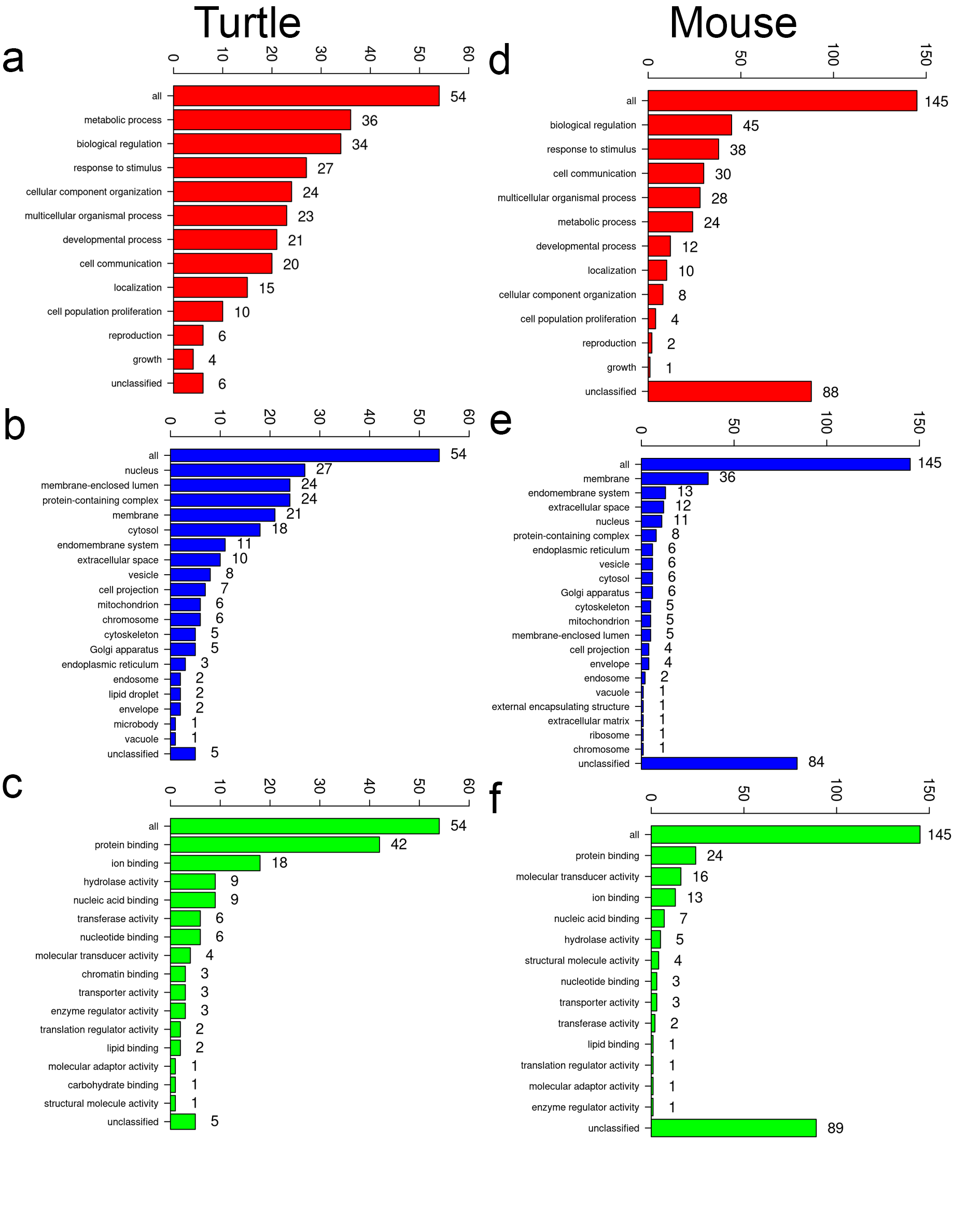

Supplement: Supplementary file 1 [file jdb-14-00016-s001.zip › Figure S2.tif]

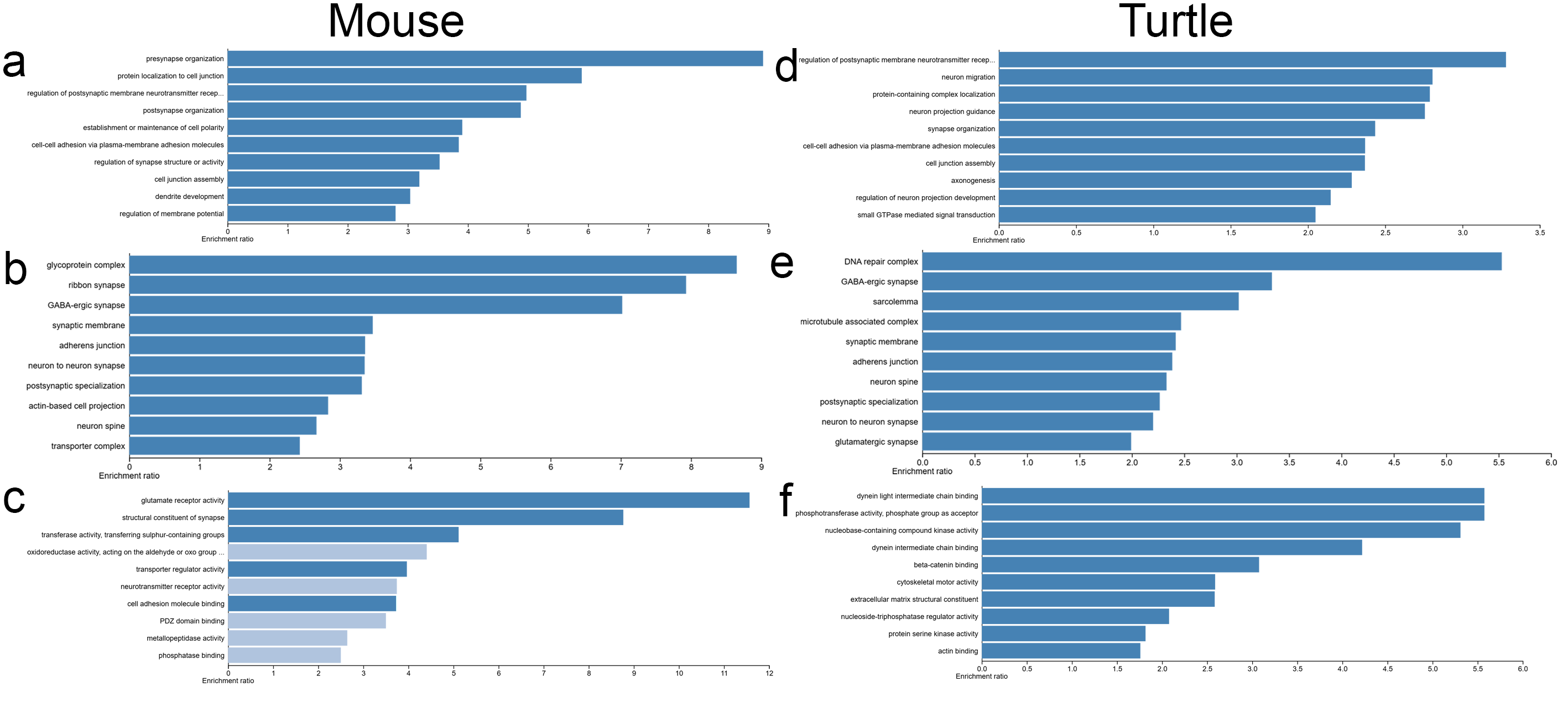

Supplement: Supplementary file 1 [file jdb-14-00016-s001.zip › Figure S3.tif]
